# Differentiated ratings of perceived exertion in upper body exercise

**DOI:** 10.1371/journal.pone.0283620

**Published:** 2023-03-24

**Authors:** Ulric S. Abonie, Marloes Oldenburg, Lucas van der Woude, Florentina J. Hettinga

**Affiliations:** 1 Department of Sport, Exercise & Rehabilitation, Northumbria University, Newcastle upon Tyne, United Kingdom; 2 Center for Human Movement Sciences and Center for Rehabilitation, University Medical Center Groningen, University of Groningen, Groningen, The Netherlands; Universidade Federal de Mato Grosso do Sul, BRAZIL

## Abstract

This study examined whether differentiated ratings of perceived exertion (RPE) (local; RPE_L_ and central; RPE_C_) and overall RPE (RPE_O_) were different between exercise modes (upper- versus lower body) and/or changed after upper body training, providing relevant input for upper body exercise prescription/regulation. Eight rowers completed an incremental cycling test (CY), and incremental handcycle (HC) tests before (HC_pre_) and after three weeks of handcycle training (HC_post_). RPEc was higher during CY (17.4±2.4) compared to HC_post_ (15.9±1.9). However, RPEo was higher during HC_post_ (9.1±0.6) compared to CY (8.3±1.1). During the HC tests, RPE_L_ was consistently higher than RPE_O_ at the same PO. Training resulted in higher RPEc (HC_pre_: 14.6±2.6; HC_post_: 15.9±1.9) and RPEo (HC_pre_: 7.9±0.9; HC_post_: 9.1±0.6). No differences were found for RPE_L_ between CY and HC_post_ (8.7±1.1; 9.3±0.4) and after HC training (HC_pre_: 9.1±1.0; HC_post_: 9.3±0.4). At the point of exhaustion, RPEc was higher in CY than during HC_pre_ and HC_post_, suggesting RPE_C_ is not causing exercise termination in HC. Furthermore, RPE_L_ is perceived higher than RPE_O_ during all stages of the incremental HC tests compared to CY. This suggests that in contrast to cycling, local factors during arm work are perceived more strongly than central or overall cues of exertion.

## Introduction

Monitoring training load can aid determining the appropriate stimulus for training adaptations by monitoring an individual’s perceived effort [1–4]. Rating of perceived exertion (RPE) provide a quantifiable measure of an individual’s subjective feeling of exertion during a physical task [4, 5]. While the use of RPE for monitoring training load has grown in popularity, much of the work related to RPE has focused on lower body endurance exercise with little focus on upper body endurance exercise [6–8]. Consequently, there is insufficient evidence regarding the reliable and valid use of RPE to understand and regulate upper body endurance exercise intensity [4, 7]. This is particularly relevant for people for whom arm work is a predominate feature in their sport, such as handcyclists, kayakists, rowers and canoeists, and could also benefit persons who rely on their upper body for activities of daily living such as wheelchair users. Until now however almost no recommendations have been made concerning the use of RPE for upper body exercise and our understanding of upper body exercise limitations is limited [7].

A greater understanding is needed, using differentiated RPE in upper body endurance exercise, to better understand perception of exertion in the upper body during endurance exercise. This could be relevant to enhance exercise motivation when prescribing exercise programs, since a relation exists between affective load and exercise engagement [4, 9]. When participants perceive an increase in sense of effort as enjoyable, they are more likely to increase or sustain their exercise behaviour. RPE could be differentiated into local (active musculature) and central (cardiorespiratory-metabolic) perceptions of exertion [10–12]. For upper body exercise, where the musculature is typically smaller [13], exertional cues from the periphery may be more pronounced compared to those from the cardiorespiratory system [14].

Literature suggests that perceptual cues may be more readily monitored from smaller muscle masses such as the upper body compared to the larger muscle masses in the lower body [5]. Borg et al. [15] observed that at higher exercise intensities there was a greater accumulation of blood lactate in the localized area of muscle activity. It is therefore reasonable to assume that a person will perceive arm exercise as requiring greater exertion than leg exercise at any given power output. Few studies have used RPE to monitor intensity during upper body exercises in a homogeneous population [7, 15, 16].

Handcycling as an alternative method of upper body exercise testing and training has received recent attention in the literature and has the potential to increase functional status and participation [16–18]. More knowledge on RPE specific to upper body training modes is required to use as input to assess and prescribe adequate exercise intensity in the upper body. The purpose of this study was to examine differentiated RPE’s (local and central) and overall RPE, and their relation to peak values of power output (PO_peak_), heart rate (HR_peak_) and oxygen uptake (VO_2peak_) during incremental exercise tests in handcycling (HC) and leg cycling (CY) in rowers (a population familiar with the Borg-scale [15], experienced in upper body endurance exercise and able to exercise at high intensity). Specifically, we were interested in whether differentiated RPE’s (local and central) and overall RPE were affected by the exercise mode. Furthermore, to better understand the impact of training on perception of exertion in upper body exercise we compared RPE before (HC_pre_) and after (HC_post_) handcycle training: do individuals report RPE differently after training? We hypothesised that local RPE would be greater during handcycling than leg cycling, and central RPE would be greater at termination of leg cycling than at termination of handcycling. Furthermore, local and central RPE would be greater after handcycling training.

## Materials and methods

### Participants

Eight trained ex-national male rowers (mean ± SD; age: 23.4 ± 2.1 years, body mass: 87.9 ± 9.2 kg, height: 1.89 ± 0.05 m.) participated in this study. Informed written consent was obtained from participants after the study rationale and procedure was explained to them, and questions they had about the study answered. Participants were initially classified as being of “high fitness” according to self-reported activity status (performing more than 5 hours of physical activity per week) and had been at professional level in the last 2 years. The participants had no previous experience with hand cycling. The study was approved by the local ethics committee, Faculty of Human Movement Sciences, University Medical Centre Groningen, Groningen, approval number ECB/26.10.2012_1, and was conducted in accordance with the Declaration of Helsinki. An a priori sample size calculation was performed using GPower (v3.0.0) using the effect size from Dallmeijer et al., [19] (Cohen’s d: 1.75) that demonstrates physiological stress and strain following handcycle. To detect the specified effect, an estimated sample size of 8 participants is required.

### Experimental design

**Fig 1:**
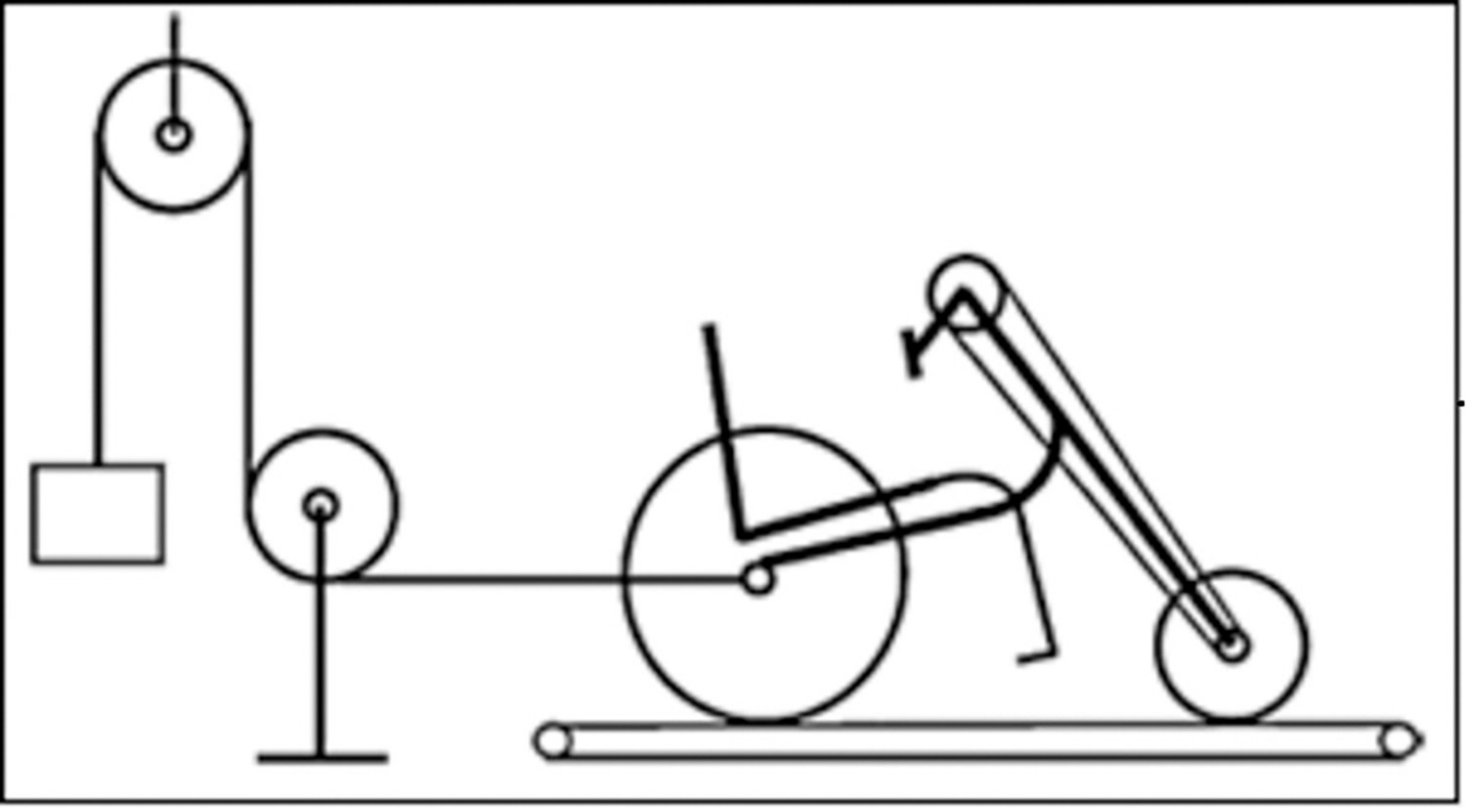
Pulley system attached to the handcycle on the treadmill.

Participants visited the laboratory on eight separate occasions in six weeks. During the first visit, participants performed an incremental exercise test on a cycle ergometer (CY). The following week, at the same time of day, participants performed an incremental exercise test on a handcycle (HC_pre_) with synchronous crank mode (Tracker Tour, Double Performance, Gouda, The Netherlands) with pulley system on a motor driven treadmill (Enraf Nonius 3446, Netherlands, belt 1.25 x 3.0 m) (Fig 1).

Before the handcycle test started, a separate drag test was performed to determine the drag force of the pulley system on different inclines using the protocol of van der Woude et al., [20]. During the HC test, exercise load was increased by adding extra weight, through a pulley system that was positioned behind the treadmill and connected to the rear wheel axle of the HC with a rope [20].

During the next five sessions, participants trained in the handcycle as used in the study of Hettinga et al., [21]. The training consisted of 15km time trials every three days for three weeks. After the three weeks, again an incremental handcycle test was performed (HC_post_).

Participants were asked to eat or intake fluid two hours before the test, wear light weight and comfortable clothes, abstain from strenuous exercise and consumption of caffeine, alcohol and salty foods. All tests were performed in a lab-controlled environment (18 ± 0.5°C, 37.2 ± 1.3%, 1021 ± 4 mmHG)

### Incremental exercise test

**Fig 2:**
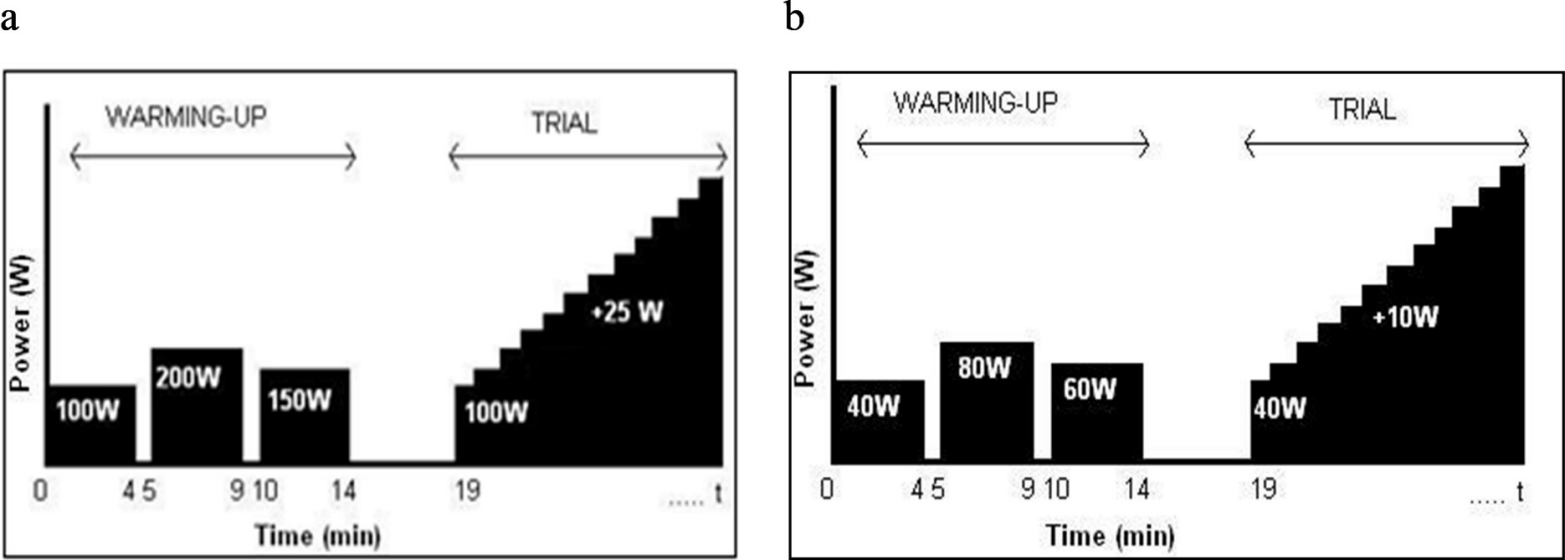
Incremental tests protocol. a. Leg Cycle test protocol and b. Handcycle test protocol.

The incremental tests were preceded by a warm-up which consisted of three 4-min constant load exercise stages at different power levels based on the study of Borg et al., [15] to get used to the propulsion and steering mechanism prior to the incremental test (Fig 2A and 2B). Protocols were chosen to reach total exhaustion and optimal VO_2_ in twelve minutes [22].

The CY protocol employed a pedal rate of 90 RPM with a starting intensity of 100W and increasing power output of 25W.min (Fig 2A). The HC protocol employed a pedal rate of 80 RPM with one-minute stepwise increase in intensity, the intensity increased 10W.min with a starting intensity of 40W [19, 23, 24] (Fig 2B). The seat configuration during HC remained constant among participants, during training and incremental tests.

All subjects were verbally encouraged to continue the exercise until volitional exhaustion. The end point of the test was determined when the participants could not maintain the expected cadence during cycling (90RPM) or handcycling (80RPM).

### Cardiorespiratory parameters

Oxygen uptake (V˙O_2_, l·min^−^^1^), carbon dioxide output (V˙CO_2_, l·min^−^^1^) and minute ventilation (V˙E, l·min−^1^) were continuously measured during the test using a computerized gas analyzing system (Oxycon Alfa, Jaeger, Bunnik, The Netherlands) using a breath-by-breath technique. Calibration of the analyzing system was performed prior to each test with reference gases (15.5% O_2_, 5.1% CO_2_) and a 3.0 l calibration syringe (Series 5530, Hans Rudolph Inc., Kansas City, MO, USA). Heart rate (HR, b·min−^1^) was measured with a heart rate monitor (Polar Sport Tester Vantage, Polar Electro Inc., Kempele, Finland), using a 5-s interval. Individual mean values of the cardiorespiratory parameters were calculated over the last 20 seconds of each exercise interval.

### Differentiated perceived exertion

The Borg 15-point RPE scale [5, 11] scale and the Borg 10-point RPE scale were used to assess ratings of perceived exertion. The Borg 15-point RPE scale ranged from 6 (no exertion at all) to 20 (maximal exertion) and was used to evaluate central perceived exertion (RPE_C,_ i.e., rating of exertion which takes into account how hard breathing feels, if the heart is pounding and/or if someone is short of breath) [5]. The Borg 10-point RPE scale ranged from 0 (nothing at all) to 10 (extremely strong) and was used to measure local (RPE_L_, i.e., how much exertion was perceived in the muscles in the arms or legs) and overall perception of exertion (RPE_O,_ i.e., whole-body) [8]. A very strong correlation (0.997–0.999) has been reported between the Borg 15-point RPE scale and the Borg 10-point RPE scale during cycling in abled-bodied men [25]. Although there is precedence in the literature for utilizing the 15-point scale for both RPE_C_ and RPE_L_, the use of two different scales (Borg 15-point RPE scale and the Borg 10-point RPE scale) allow to differentiate the relative contribution of one mechanism over another [15, 25].

Participants were given standardized instructions on how to report RPE_C_, RPE_L_ and RPE_O_ during leg and arm exercise. In the last 15 seconds of each minute interval participants were asked to appraise their RPE_C_, RPE_L_ and RPE_O_. The participants indicated a RPE value by using either a finger signal or head movement in response to prompts by the investigator.

### Statistical analyses

All data were analysed using the statistical package SPSS for windows version 20 (SPSS for Windows Version 16.0; SPSS, Inc, Chicago, IL). All data were presented as mean ± standard deviation. Series of non-parametric Friedman tests and Wilcoxon signed-rank tests were used to compare differences between RPE_L_, RPE_C_ and RPE_O_ at 1). the termination of the CY versus the HC_post_ and 2). between HC_pre_ and HC_post_ exercise.

Pearson’s and Spearman’s product correlation analysis were performed to analyse the relationships between RPEs, peak heart rate (HR_peak_), peak oxygen uptake (VO_2peak_), lactate concentration (BLa^-^), peak minute ventilation (VE_peak_), carbon dioxide output (VCO_2_), and peak power output (PO_peak_) during cycling and handcycling. Individual R^2^ values were obtained for each participant to identify the relationship between RPE_L_, RPE_C_ and RPE_O_ and each physiologic marker of exercise intensity (HR, VO_2_). Level of significance was set at 0.05.

## Results

### Descriptive statistics

All peak physiological responses and RPEs elicited at the termination of the cycling and handcycling tests are shown in Table 1.

**Table 1:** Mean peak outcomes of incremental maximal cycle (CY), handcycle before (HC_pre_) and after (HC_post_) training.

N = 8	CY (mean ± SD)	HC_pre_ (mean ± SD)	HC_post_ (mean ± SD)
Time to exhaustion (min)	13.1 ± 1.2	10.7 ± 1.5	11.7 ± 1.2 ^α^^,^^β^
VO_2peak_ (Litres.min^-1^)	4.92 ± 0.29	2.91 ± 0.34	3.14 ± 0.46 ^α^^,^^β^
V_Epeak_ (Litres.min^-1^)	1.82 ± 0.25	0.92 ± 0.18	1.05 ± 0.22^α^^,^^β^
HR _peak_ (beats.min^-1^)	194 ± 10	181 ± 13	182 ± 10^α^
RER	1.23 ± 0.06	1.18 ± 0.09	1.21 ± 0.09
PO_peak_ (W)	425 ± 25	141 ± 16	152 ± 20^α^^,^^β^
BLa^-1^ (mmol.L^-1^)	12.3 ± 1.6	9.2 ± 2.5	10.6 ± 1.7^α^
RPE_O_	8.3 ± 1.1	7.9 ± 0.9	9.1 ± 0.6^α^^,^^β^
RPE_L_	8.7 ± 1.1	9.1 ± 1.0	9.3 ± 0.4
RPE_C_	17.4 ± 2.4	14.6 ± 2.6	15.9 ± 1.9^α^^,^^β^

VO_2peak_: peak oxygen uptake, VE_peak_: peak minute ventilation, HR_peak_: peak heart rate, RER: respiratory exchange ratio, PO_peak_: peak power output, BLa^-1^: blood lactate, RPE_O_: overall rating of perceived exertion, ranging from 0 to 10, RPE_L_: local rating of perceived exertion ranging from 0 to 10, RPE_C_: central rating of perceived exertion ranging from 6 to 20.

α. Significant at the P < 0.05 level between CY and HC_post_

β. Significant at the P < 0.05 level between HC_pre_ and HC_post_

Paired t-tests comparing peak values in CY with HC_post_, revealed significant differences for time to exhaustion (p = 0.005), HR_peak_ (p = 0.002), VO_2peak_ (p = 0.002), BLa^-1^ (p = 0.027) and PO_peak_, (p < 0.001).

The VO_2peak_ HR_peak_, PO_peak_ and BLa^-1^ during handcycling were 67 ± 9%, 94 ± 2%, 32 ± 13% and 85 ± 12% respectively of the corresponding values during cycling.

Between HC_pre_ and HC_post_, significant differences were observed for time to exhaustion (p = .008), VO_2peak_ (p = 0.027) and PO_peak_ (p = 0.003). No differences were found for HR_peak_, VE_peak_, BLa^-^ and respiratory exchange ratio (RER) (p > 0.05).

### RPE and physiologic markers

Pearson Product correlation showed very strong relationships between HR_peak_ and VO_2peak_ in all exercise modes (CY: *r* = 0.990, p < 0.001; HC_pre_: *r* = 0.970, p < 0.001; HC_post_: *r* = 0.996, p < 0.001).

Table 2 shows the Spearman Product-Moment correlations of RPE’s and physiologic markers for the sample. All RPEs related linearly to HR _peak_, VO_2 peak_ and PO_peak_ during the CY and HC tests (p < .001). Linear regression analyses of individual RPEs and VO_2peak_, RPEs and HR_peak_, RPEs and PO_peak_ produced average R^2^ values of R^2^ = 0.94.

**Table 2:** Spearman correlation coefficients of RPEs and cardiorespiratory markers during cycle, and before and after handcycle training.

	HR_peak_	VO_2peak_	PO_peak_
Cycle (CY)			
RPE_L_	0.988*	0.983*	0.995*
RPE_O_	0.985*	0.984*	0.993*
RPE_C_	0.986*	0.979*	0.991*
Handcycle before training (HC_pre_)			
RPE_L_	0.950*	0.953*	0.985*
RPE_O_	0.966*	0.973*	0.975*
RPE_C_	0.939*	0.961*	0.948*
Handcycle after training (HC_post_)			
RPE_L_	0.993*	0.985*	0.988*
RPE_O_	0.971*	0.971*	0.971*
RPE_C_	0.986*	0.988*	0.992*

HR_peak_: peak heart rate, VO_2peak_: peak oxygen uptake, PO_peak_: peak power output, RPE_O_: overall rating of perceived exertion, ranging from 0 to 10, RPE_L_: local rating of perceived exertion ranging from 0 to 10, RPE_C_: central rating of perceived exertion ranging from 6 to 20.

*Significant relationship between variables (p < 0.05).

### Differentiated RPE’s

All absolute RPEs were perceived significantly (p < 0.001) higher during arm than leg exercise at any given PO, HR or VO_2_ throughout the test. This contrasts with the peak RPE values at exhaustion. RPE_C_ was lesser in HC_post_ compared to CY (p = 0.039). Also, RPE_O_ was significantly greater during HC_post_ than CY (p = .046). No differences were observed for RPE_L_ between exercise modes (CY versus HC_post_; p = 0.088) and before and after training (HC_pre_ versus HC_post_; p = 0.336). After three weeks of training, differences were found between HC_pre_ and HC_post_ for RPE_C_ (HC_pre_: 14.6 ± 2.6; HC_post_: 15.9 ± 1.9; p = 0.046) and RPE_O_ (HC_pre_: 7.9 ± 0.9; HC_post_: 9.1 ± 0.6; p = 0.026).

**Fig 3:**
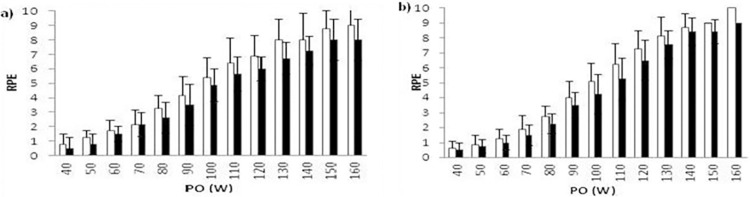
Local (white bar) and overall (black bar) perceived exertion during. a) handcycling before training and b) handcycling after training.

Finally, RPE_L_ seems to be perceived heavier than RPE_O_ throughout the entire incremental HC test. As presented in Fig 3, there was a tendency for sensations of local muscle fatigue in the arms that seemed to be more severe and earlier in the test than overall effort of perception (HC_pre_, p = 0.049; HC_post_, p = 0.32).

## Discussion

This study examined local, central and overall RPE, and their relation to peak values of power output (PO_peak_), heart rate (HR_peak_) and oxygen uptake (VO_2peak_) during incremental exercise tests in hand cycling (HC) and leg cycling in rowers. The most striking outcomes of the study were that all absolute RPEs (local, central and overall) were perceived higher during arm compared to leg exercise at any given PO, HR or VO_2_ and RPE increased linearly with PO_peak_, HR_peak_ and VO_2peak_. This provides insight into the challenges of upper body endurance exercise, and potential use of RPE to monitor and regulate upper body endurance exercise intensity.

The higher absolute RPE´s (local, central and overall) during arm compared to leg exercise at any given PO, HR or VO_2_ throughout the entire incremental test found in this study could be explained by the difference of muscle mass between the arms and legs [10]. To maintain the same power output with a smaller muscle mass, the participant has to work comparatively harder with the arms than with the legs [13]. As a result, blood flow increases, lactate rises and, most important, participants thus perceive a higher degree of exertion [10, 15]. The strong positive linear relationship between RPE and PO_peak_, HR_peak_ and VO_2peak_ found in this study suggest that the RPE may be a valuable and useful tool to monitor and regulate upper body exercise intensities in (adapted) sports and rehabilitation settings [7, 26].

Furthermore, participants reported higher RPE_L_, perceiving fatigue, aches and pains in the legs or arms to a greater extent than whole-body ratings of exertion. Previous studies that explored the use of RPE in regulating upper body exercise did not differentiate local and central RPE and or did evaluate in relation to lower body exercise [7, 15]. Examining differentiated RPE´s during CY and HC, we see that at the termination of the CY test, peak RPE_C_ was reported higher compared to HC. This is because central cues, such as HR_peak_ and VO_2peak_, dominate one’s perception of exertion at higher intensities which can easier be reached during CY compared to HC [10]. This agrees with the results of the current study that showed differences between arm and leg exercise in peak physiological variables at absolute levels of intensity. The observed higher values for VO_2peak_, HR_peak_, Bla^-1^, RER, PO_peak_ and VE_peak_ during CY compared to HC are comparable to previous studies that used arm crank and upper body poling exercise mode [10, 11]. Central factors thus seem to play a smaller role in endurance activity limitations than has been previously suggested [27]. This current study indicates that exercise limitations of upper body endurance exercise are not central but more at local level.

An unexpected finding in this study was that there were no differences found at exhaustion for RPE_L_ between CY and HC. This is contrary to earlier evidence of a higher relative RPE_L_ in arm compared to leg exercise [10, 15]. Pandolf et al., [11] reported that the effect of exercise time on rated exertion was higher at longer test durations. An increase of lactic acidic in working muscles has been evident to signals RPE_L_ [4]. The lack of a significant difference in RPE_L_ between CY and HC may thus be explained by the longer CY test duration compared to HC. Indeed, examination of our results (see Table 1) shows that time duration and blood lactate were higher during CY compared to HC. However, RPE_L_ was consistently reported higher than RPEo at the same PO pre- and post-tests, suggesting that RPE_L_ plays a larger role in arm than leg exercise. Additionally, RPE_O_ was reported higher during HC compared to CY.

The secondary purpose of this study was to examine the effect of HC training on the ratings of perceived exertion (RPE) to better understand the impact of training on perception of exertion in upper body exercise. We observed that RPE_O_ and RPE_C_ were affected by training. This is caused by the fact that participants reached higher PO_peak_ in the HC_post_ compared to the HC_pre_. As mentioned before, higher RPEc scores are a consequence of central factors that dominates the perception of exertion at higher intensities. Therefore, it is not surprising that participants reported higher RPEc values after HC training, indicating training leads to higher physiological responses and thus may have cardiorespiratory benefit.

Exploring utility of differentiated RPE in able-bodied rowers (experienced in upper body exercise) is the first step to examine RPE as an appropriate tool to estimate and prescribe upper body endurance exercise intensity. A particular population that could benefit from the results of this study is wheelchair users, for example people with a spinal cord injury (SCI). Validation of these findings in individuals with a SCI is then warranted and future research studies should focus on discriminating local from central cues to establish a method for using RPE as a valid and reliable indicator of exertion in persons with SCI and extend these findings to activities of daily living or more practical rehabilitation-based sessions to estimate and prescribe appropriate activities.

## Conclusion

This study has shown encouraging potential for the use of RPE in monitoring and prescribing appropriate upper body exercise for people who are interested in improving upper body performance and could be a plausible tool to facilitate positive affect such as enjoyment and motivation, or reduce negative affect associated with perceptions of exertion, which can lead to increase exercise behaviour. The study examined whether differentiated RPE’s (local, central or overall) were affected by exercise mode (upper- versus lower body) and showed that local RPE provided the dominant perceptual signals during upper body exercise and central RPE provided the dominant perceptual signals during cycling. Furthermore, the study suggests that the equivocality in previous research can be explained by the importance of local factors.

In support with past studies RPE_C_ and RPE_O_ were significantly greater and lesser respectively during cycling compared to handcycling at any given power output, heart rate or oxygen uptake. Higher physiological variables were reached at any given power output in cycling compared to handcycling. Furthermore, at any given power output, ratings of perceived exertion were higher during cycling compared to handcycling and were linearly related to physiological variables.
